# Recurrent Small Bowel Obstruction in an Adult Male Patient Due to Complicated Meckel’s Diverticulum: A Case Report

**DOI:** 10.7759/cureus.108072

**Published:** 2026-04-30

**Authors:** Hugo E Mora Moreno, Roberto Gomez Zamayoa, Gerardo Galvez Estrada, Omar J Madrigal Flores, Eduardo J Hernandez Mendoza

**Affiliations:** 1 General Surgery, Hospital General Dr. Miguel Silva, Morelia, MEX

**Keywords:** ileal diverticulum, meckel’s diverticulum, recurrent small bowel obstruction, small bowel obstruction, surgical management

## Abstract

Meckel’s diverticulum is the most common congenital anomaly of the gastrointestinal tract, although it remains asymptomatic in most cases and is rarely diagnosed in adulthood. When symptomatic, it may present with complications such as intestinal obstruction, which can pose a diagnostic challenge due to its nonspecific clinical features. We report the case of a 31-year-old man with a history of multiple prior episodes of small bowel obstruction managed conservatively without an established etiology, who presented with a five-day history of progressive abdominal pain, abdominal distension, non-bilious vomiting, and constipation. Laboratory studies revealed electrolyte imbalances, while contrast-enhanced computed tomography demonstrated a blind-ending structure arising from the distal ileum with associated small bowel dilation and a transition point, suggestive of Meckel’s diverticulum. The patient underwent exploratory laparotomy, which confirmed an inflamed Meckel’s diverticulum acting as the cause of mechanical obstruction. A wedge resection was performed with an uneventful postoperative course. Histopathological analysis confirmed the diagnosis. This case highlights the importance of considering Meckel’s diverticulum in the differential diagnosis of recurrent small bowel obstruction in young adults, particularly in patients without prior abdominal surgery, as early recognition and surgical management are essential to prevent complications and ensure optimal outcomes.

## Introduction

Meckel’s diverticulum is the most common congenital anomaly of the gastrointestinal tract, resulting from incomplete obliteration of the omphalomesenteric duct. Although present in approximately 2%-4% of the population, the majority of cases remain asymptomatic and are often incidentally discovered during imaging or surgical procedures [1,2].

Symptomatic presentations are relatively uncommon in adults and can pose a diagnostic challenge due to their nonspecific clinical manifestations. When complications occur, intestinal obstruction is one of the most frequent presentations in the adult population, followed by inflammation, bleeding, and, less commonly, perforation [3]. The mechanism of obstruction may include inflammatory adhesions, volvulus around fibrous bands, intussusception, or incarceration of the diverticulum, often mimicking other acute abdominal conditions [2,4].

Preoperative diagnosis remains difficult despite advances in imaging modalities. Computed tomography (CT) may reveal suggestive findings such as a blind-ending structure arising from the antimesenteric border of the ileum or the presence of a characteristic feeding vessel; however, these findings are not always readily identifiable, contributing to delayed or missed diagnosis [1,4].

Given its variable presentation and potential for recurrent or acute complications, Meckel’s diverticulum should be considered in the differential diagnosis of small bowel obstruction, particularly in young adults without prior abdominal surgery or with recurrent obstructive episodes of unclear etiology. Early recognition of suggestive imaging findings and correlation with the clinical course may help guide timely surgical decision-making. We present a case of recurrent small bowel obstruction in a young adult caused by a complicated Meckel’s diverticulum requiring surgical management.

## Case presentation

A 31-year-old man with a history of recurrent obstructive episodes suggestive of small bowel obstruction (SBO), including at least five prior episodes managed conservatively at outside institutions without a definitive etiological diagnosis, presented to the emergency department with a five-day history of progressively worsening colicky abdominal pain. The current episode was associated with marked abdominal distension, nausea, multiple episodes of non-bilious vomiting, and absence of bowel movements and flatus.

On initial evaluation, the patient appeared mildly dehydrated but hemodynamically stable. Abdominal examination revealed significant distension, decreased bowel sounds, and generalized tympany, with diffuse tenderness to deep palpation but no signs of peritoneal irritation. Hernial orifices were intact, with no evidence of external hernias or prior surgical scars.

Laboratory evaluation demonstrated electrolyte abnormalities, including hyponatremia (129 mmol/L) and hypochloremia (86 mmol/L), with normal potassium levels (3.74 mmol/L). The white blood cell count and C-reactive protein were within normal limits (Table 1).

**Table 1:** Laboratory findings on admission. Laboratory values obtained at the time of initial presentation to the emergency department, demonstrating electrolyte abnormalities consistent with dehydration secondary to small bowel obstruction.

Test	Result	Reference Range
Sodium	129 mmol/L	135-145 mmol/L
Chloride	86 mmol/L	98-107 mmol/L
Potassium	3.74 mmol/L	3.5-5.0 mmol/L
White Blood Cells	5.7 ×10³/µL	4.5-10×10³/µL
C-Reactive Protein	0.8 mg/L	0-6 mg/L

**Figure 1:**
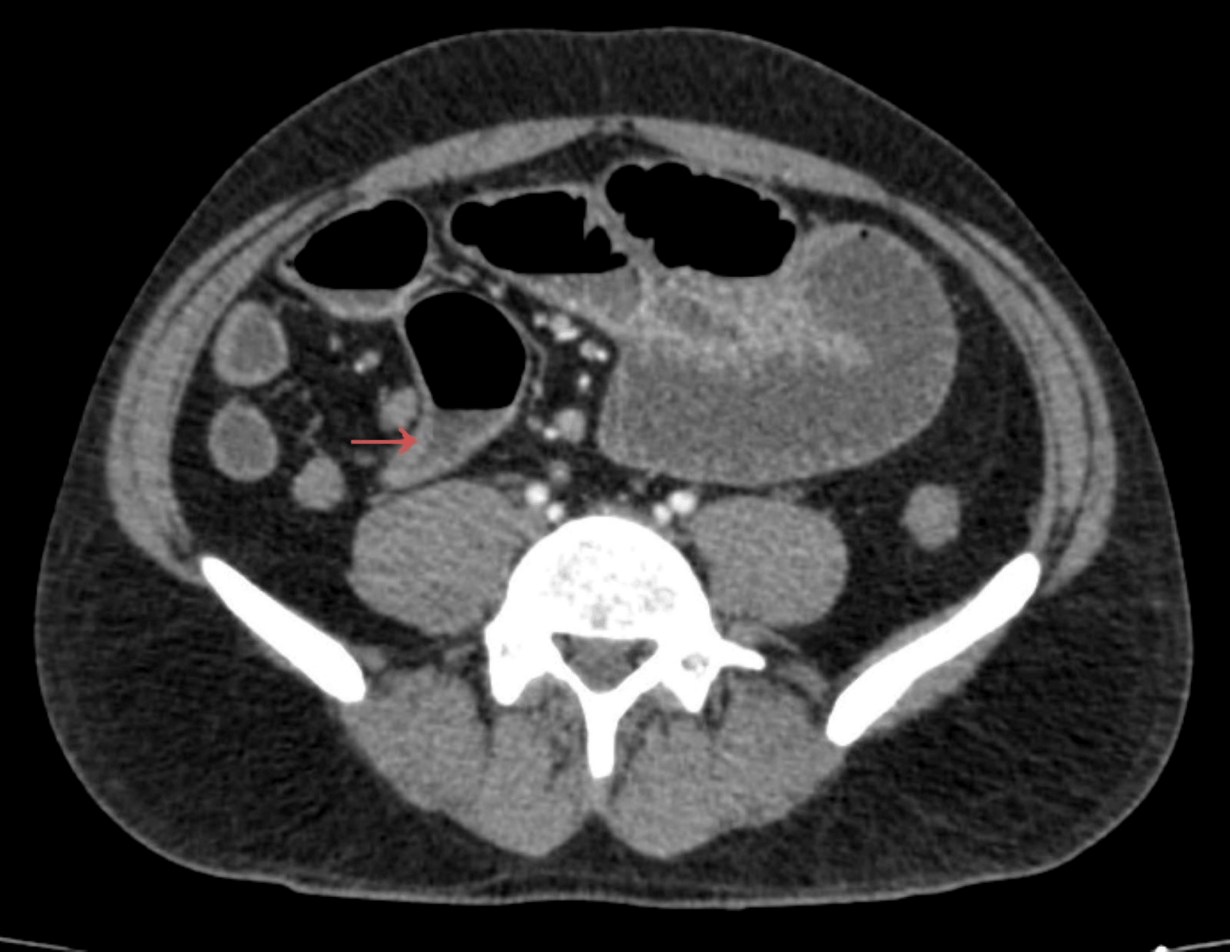
Axial contrast-enhanced CT showing Meckel’s diverticulum. Axial contrast-enhanced computed tomography (CT) image demonstrating a blind-ending, sac-like structure arising from the antimesenteric border of the terminal ileum (arrow), containing intraluminal material, consistent with Meckel’s diverticulum.

**Figure 2:**
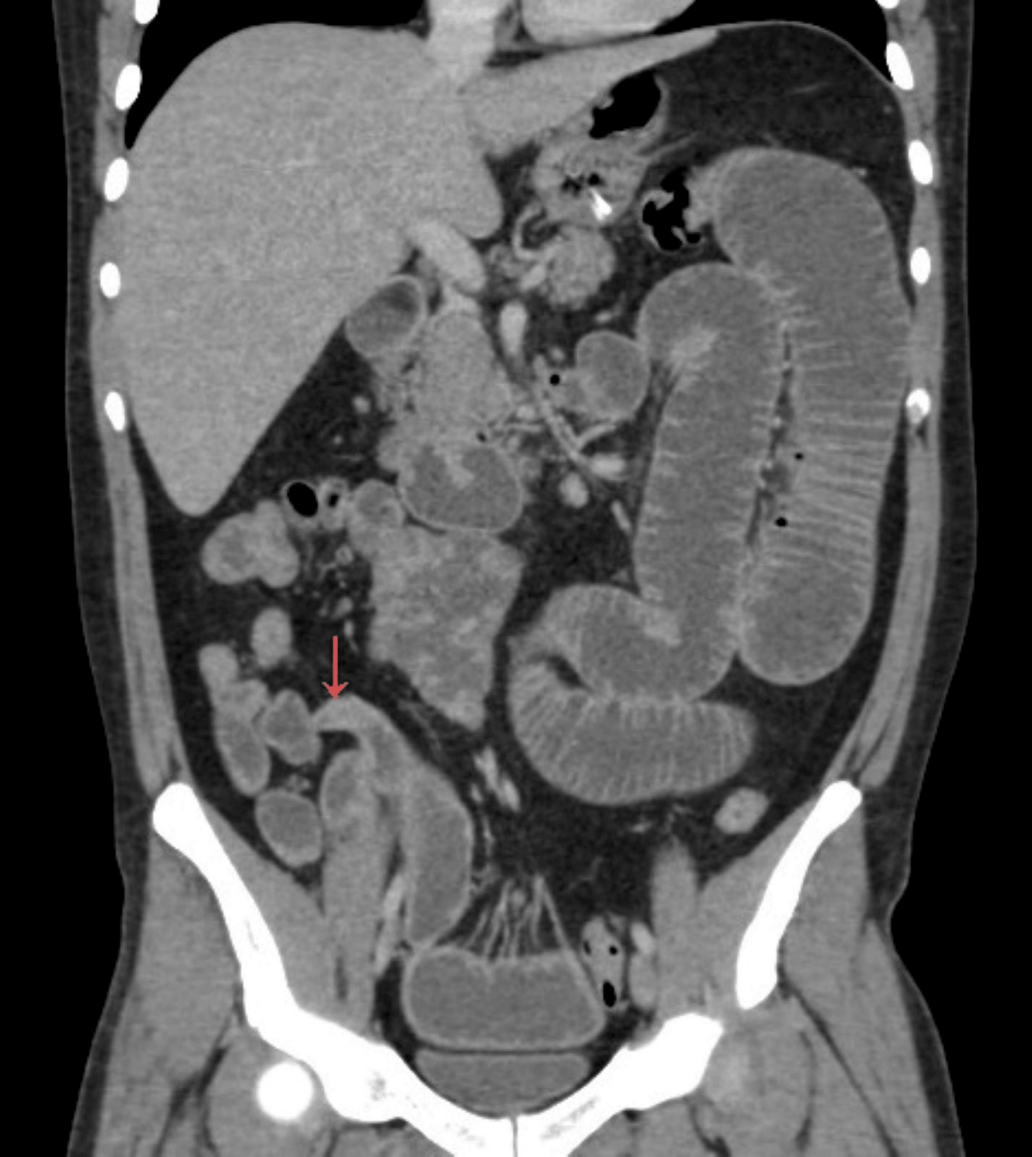
Coronal contrast-enhanced CT reconstruction of Meckel’s diverticulum. Coronal contrast-enhanced CT image demonstrating a blind-ending diverticular structure arising from the antimesenteric border of the distal ileum (arrow), consistent with Meckel’s diverticulum, with improved visualization of its anatomical orientation.

**Figure 3:**
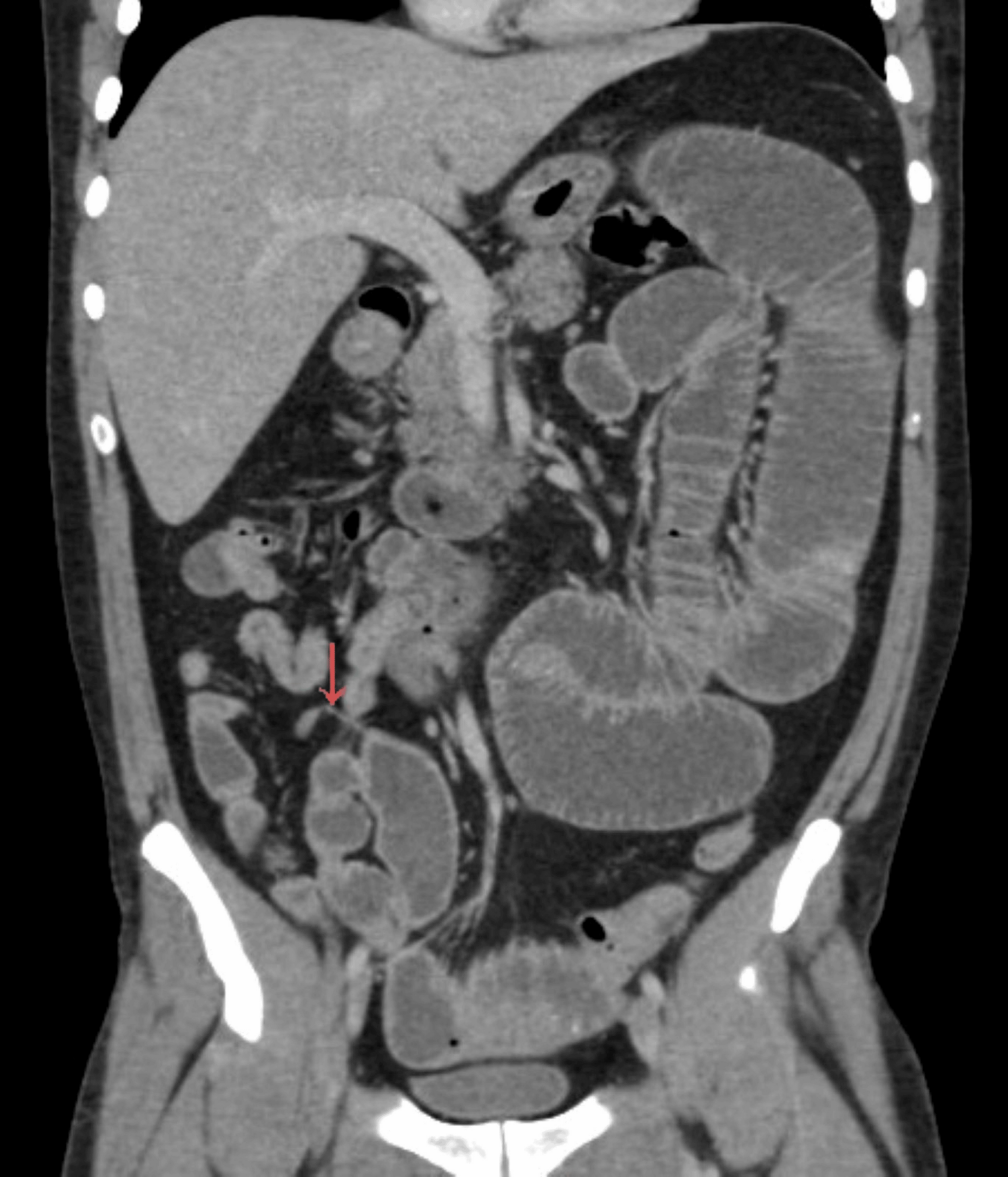
CT identification of a feeding vessel associated with Meckel’s diverticulum. Contrast-enhanced CT image showing a prominent vessel supplying the diverticulum (arrow), a radiologic feature that supports the diagnosis of Meckel’s diverticulum.

Contrast-enhanced computed tomography (CT) of the abdomen and pelvis demonstrated dilated small bowel loops with multiple air-fluid levels and a well-defined transition point in the distal ileum. A blind-ending, sac-like structure arising from the antimesenteric border of the ileum was also identified (Figures 1, 2), containing intraluminal material and associated with a prominent feeding vessel (Figure 3). These findings were highly suggestive of Meckel’s diverticulum.

Given the persistence of obstructive symptoms and radiologic findings consistent with mechanical SBO, the patient was taken to the operating room for urgent exploratory laparotomy.

**Figure 4:**
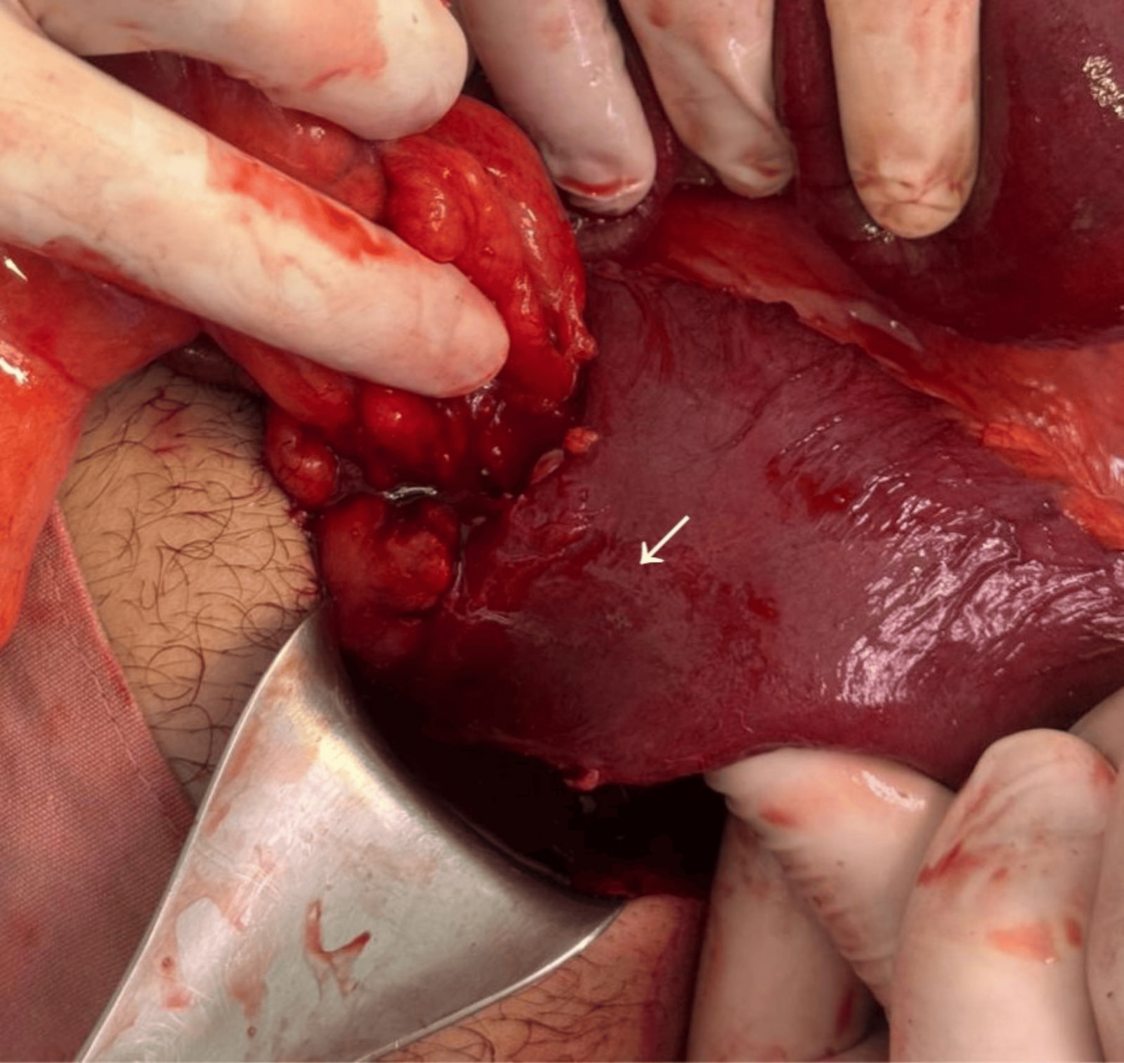
Inflamed Meckel’s diverticulum causing bowel obstruction. Intraoperative image demonstrating an inflamed segment of distal ileum with an adherent Meckel’s diverticulum (arrow), forming an inflammatory mass responsible for mechanical bowel obstruction.

**Figure 5:**
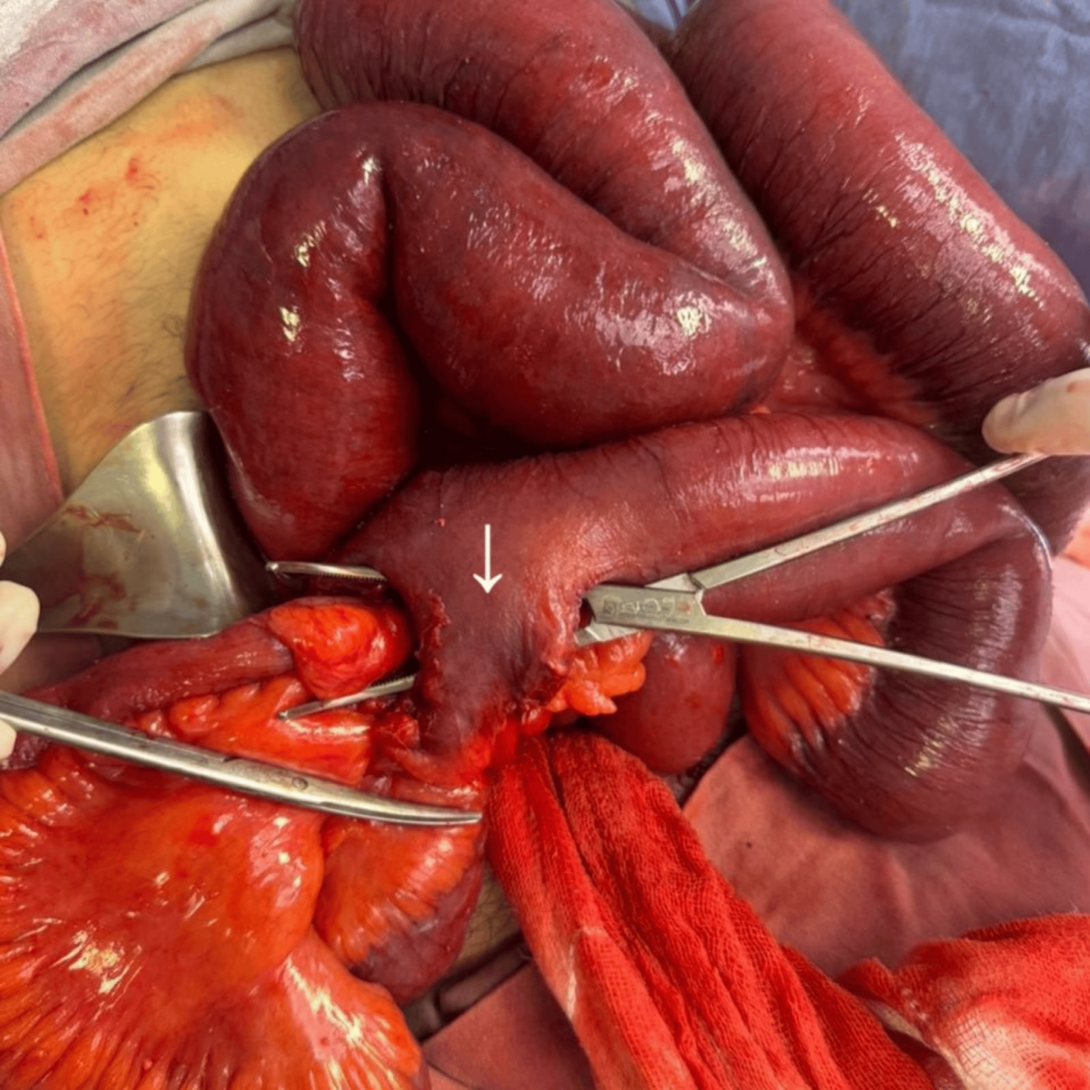
Meckel’s diverticulum with surrounding inflammatory adhesions. Intraoperative view showing a Meckel’s diverticulum (arrow) arising from the antimesenteric border of the ileum, with surrounding inflammatory adhesions contributing to luminal narrowing and obstruction.

**Figure 6:**
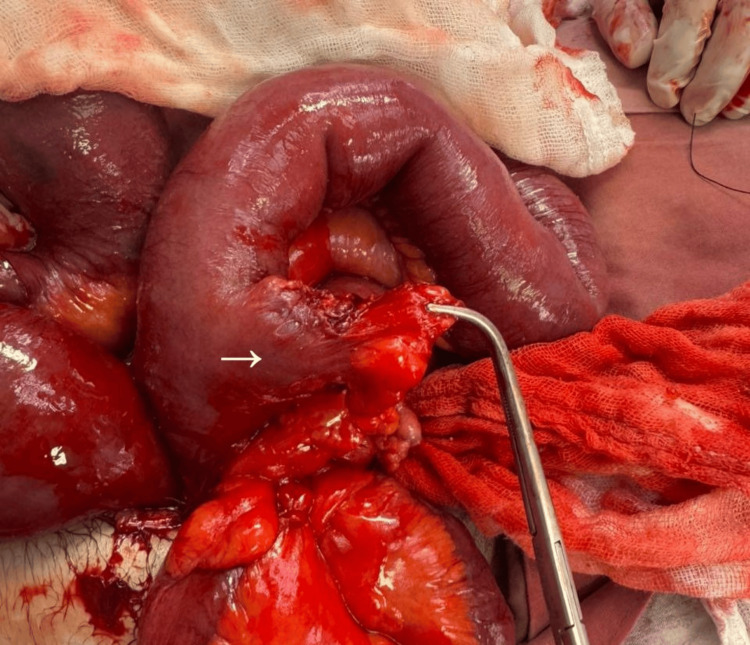
Meckel’s diverticulum as the transition point after dissection. Intraoperative image obtained after dissection, highlighting the Meckel’s diverticulum (arrow) as the definitive transition point responsible for mechanical small bowel obstruction.

Intraoperatively, an inflammatory conglomerate involving the distal ileum approximately 60 cm proximal to the ileocecal valve was identified, with significant proximal bowel dilation. A Meckel’s diverticulum was identified within the inflammatory process and acted as the lead point for intussusception, resulting in mechanical SBO (Figures 4-6). No additional intra-abdominal pathology was identified.

A wedge resection of the diverticulum was performed, preserving bowel continuity and achieving adequate hemostasis. Segmental small bowel resection and anastomosis were not required. A closed-suction drain was placed adjacent to the ileal resection site. The procedure was completed without intraoperative complications.

Postoperatively, the patient had an uneventful recovery. He was initially managed with bowel rest, followed by a gradual return of bowel function, including passage of flatus and bowel movements within the first few postoperative days. Oral intake was gradually resumed 72 hours after surgery and was well tolerated. The closed-suction drain showed minimal serous output, and no surgical site infection or other postoperative complications were observed.

The patient was discharged in stable condition on postoperative day 7, following complete resolution of obstructive symptoms and adequate tolerance of oral intake.

At one-month follow-up, the patient remained asymptomatic, with normal bowel function and appropriate wound healing. Histopathological examination confirmed a Meckel’s diverticulum measuring 4.3×3.2×1.7 cm, with features of chronic inflammation and vascular congestion.

## Discussion

Meckel’s diverticulum is an uncommon but clinically significant cause of SBO in adults, often posing a diagnostic challenge due to its variable presentation and low preoperative detection rate. While most cases remain asymptomatic, symptomatic presentations in adults are rare and frequently associated with complications, among which intestinal obstruction is the most common [5-7].

The pathophysiology of obstruction in Meckel’s diverticulum is multifactorial. Reported mechanisms include inflammatory adhesions, volvulus around fibrous bands, internal herniation, mesodiverticular bands, intussusception, and incarceration of the diverticulum, all of which may lead to luminal compromise and acute or recurrent obstruction [6-9]. In our case, the diverticulum acted as the lead point for intussusception, resulting in mechanical small bowel obstruction. This mechanism is consistent with previously described causes of Meckel’s diverticulum-related obstruction in the literature [6-9].

A notable feature of this case is the history of recurrent small bowel obstruction without prior abdominal surgery, often referred to as a “virgin abdomen.” In such scenarios, Meckel’s diverticulum should be strongly considered as an underlying etiology, particularly in young patients [7,10]. Similar cases have demonstrated that recurrent, self-limited obstructive episodes may precede definitive diagnosis, as seen in our patient, who had multiple prior conservatively managed episodes before surgical intervention [6,8].

Preoperative diagnosis remains challenging despite advancements in imaging. Computed tomography is the most widely used modality in acute settings; however, its sensitivity for detecting Meckel’s diverticulum varies significantly. Typical findings include a blind-ending structure arising from the antimesenteric border and, in some cases, a feeding vessel, although these features may be subtle or overlooked [2,8]. In this case, CT imaging successfully identified both the diverticular structure and its vascular supply, facilitating timely surgical decision-making.

Surgical management remains the definitive treatment for symptomatic Meckel’s diverticulum. The choice of technique depends on intraoperative findings, including the presence of inflammation, ischemia, or associated complications. Options include diverticulectomy, wedge resection, or segmental bowel resection [3,5]. In our patient, a wedge resection was sufficient due to localized disease without evidence of extensive bowel compromise, aligning with current surgical recommendations.

The management of incidentally discovered Meckel’s diverticulum remains controversial; however, surgical resection is widely recommended in symptomatic cases, as in our patient, given the increased risk of recurrent complications.

An important consideration is the potential morbidity associated with delayed diagnosis. Recurrent obstruction, inflammation, and risk of ischemia or perforation underscore the importance of early surgical intervention in symptomatic patients [6]. Literature suggests that prompt operative management leads to favorable outcomes with low complication rates, as observed in this case, where the patient had an uneventful postoperative recovery.

This case highlights the importance of maintaining a high index of suspicion for Meckel’s diverticulum in young adults presenting with recurrent small bowel obstruction, particularly in the absence of prior surgical history. Early recognition and timely surgical management are essential to prevent complications and improve patient outcomes.

## Conclusions

Meckel’s diverticulum should be considered an important differential diagnosis in young adults presenting with recurrent SBO, particularly in the absence of prior abdominal surgery. Its variable and often nonspecific clinical presentation contributes to delayed diagnosis, emphasizing the role of imaging and a high index of suspicion. This case highlights that recurrent, self-limited obstructive episodes may precede definitive identification of the underlying pathology. Surgical intervention remains the definitive treatment in symptomatic patients, with favorable outcomes when performed in a timely manner. Early recognition and appropriate management are essential to prevent complications and improve patient prognosis.
